# Supplementary material for: Are carbon emissions trading and green financial instruments synergistic? -Comprehensive quantitative research based on content analysis
Source: PLoS One. 2024 Mar 7;19(3):e0298601. doi: 10.1371/journal.pone.0298601 (PMC10919601; doi:10.1371/journal.pone.0298601)
Supplement: S1 Data — (ZIP) [file pone.0298601.s001.zip › Data/地级市Z_F工作报告.zip!/╕ú╜¿/╒─╓▌╩╨/└┤╫╘╣1⁄2╓┌║┼í╛┬φ┐╦╩2╛▌═°í┐_2008.docx]

各位代表：

现在，我代表漳州市人民政府向大会作工作报告，请予审议，并请市政协委员和其他列席人员提出意见。

一、2007年工作回顾

过去的一年，在市委的正确领导下，在市人大法律监督、工作监督和市政协民主监督下，我市各级政府坚持以邓小平理论和“三个代表”重要思想为指导，全面落实科学发展观，认真贯彻党的十七大精神，按照市第九次党代会和市委九届五次全会的决策部署，积极参与海峡西岸经济区建设，大力实施“依港立市、工业强市、开放活市、科教兴市”发展战略，顺利完成市十四届人大一次会议确定的各项目标任务，全市经济社会发展呈现速度加快、后劲增强、效益提升、民生改善的良好态势。

（一）国民经济快速增长，综合实力稳步提升

经济发展提速增效。实现生产总值863.73亿元，增长15.2%，增幅创1995年以来新高。三次产业结构由23.5∶42.4∶34.1调整为22.6∶43.1∶34.3。实现财政总收入83.84亿元，增长31.5%，其中地方级收入47.41 亿元，增长35.2%。财政总收入占GDP比重9.7%，提高0.8个百分点。

工业经济效益提升。完成工业总产值1212.59亿元，增长23.5%，其中规模工业产值1001.8 亿元，突破千亿元大关，增长25.5%。工业经济对GDP增长贡献率达49.2%，工业税收占税收收入比重达51.4%。“千百工程”超额完成，规模工业企业达1604 家，新增321 家，其中产值上亿元企业186 家，净增53 家。产业集聚度进一步提高，“3+4”产业产值790.9亿元，增长19.1%，其中钢铁、汽车、船舶分别增长21.8%、45.3%和55.4%。工业经济效益综合指数156.5，创历史最好水平。新增6件中国驰名商标、1件中国名牌产品和2件国家免检产品。

现代农业稳步增长。实现农业总产值343.07亿元，增长5.7%。其中，渔业产值117.01亿元，增长6.9%；花卉业销售额13.92亿元、出口额1710万美元，分别增长10%、20%。农产品出口11.8亿美元，增长20.8%。建立117个现代农业示范基地、57个专业乡镇（场）和156个产业专业村，新创办农村经济合作组织46家。平和?溪蜜柚荣获中国驰名商标，天宝香蕉、云霄枇杷获得国家地理标志产品保护，新增20个“绿标”产品、39个无公害产品。市农业检验监测中心被确定为全国绿色食品定点监测机构。

第三产业加快发展。实现第三产业增加值296.3亿元，增长17.2%；社会消费品零售总额302.28亿元，增长17%，均创1999年以来新高。建成东山水产品批发市场、华安仙都茶叶市场等一批商贸物流项目，开工建设4家五星级大酒店，滨海火山地质公园被评为国家4A级旅游区。金融支持发展力度加大，年末银行机构本外币贷款余额440.51亿元，增长27.4%。港口货物吞吐量2832.35万吨，增长26.4%，其中集装箱吞吐量26.78万标箱，增长41.1%。

（二）改革开放持续深化，发展活力日益增强

体制改革纵深推进。公交公司与长运集团完成合并，福糖公司完成改制，东区污水处理厂实现产业化运营，九龙岭垃圾处理场特许经营权成功转让。新一轮行政审批制度改革公布实施，行政服务中心重建和公共资源交易中心组建顺利完成，公共资源市场化配置改革扎实推进。林权制度改革深入实施，在全省率先开展海域使用权招拍挂和抵押贷款。银行业支持新农村信贷综合试点取得阶段性成效，农村信用社改革初步完成。

对外开放有效拓展。新批注册合同外资9.4亿美元，增长7.3%；验资到资4.5亿美元，增长12.4%。外资企业增资120家，增资额2.28亿美元，增长128.4%。外贸出口34.17亿美元，增长14.6%。新批台资项目163个，合同台资4.69亿美元，增长75.4%。台湾农民创业园已落户台资农业企业62家，总投资近亿美元，实现产值15亿元。继金门之后又与澎湖实现货运直航。来漳台胞大幅增加，签注人次21000多人次，增长近10倍。海峡两岸（福建东山）水产品加工集散基地正式获批。

县域经济活力增强。各县立足本地特色相继举办节会活动，发展氛围进一步造浓。八县规模工业增加值、财政总收入、城镇以上投资增长速度分别高出全市平均水平7.5、4.2和4个百分点，呈现竞相发展的良好势头。龙海市连续13年保持福建县域经济实力十强，长泰、华安进入福建县域经济发展十佳。

民营经济支撑有力。民营工业实现产值334.02亿元，增长48.3%。城镇以上民间投资完成125.28亿元，占城镇固定资产投资比重达44.8%，增长65.5%。217家民营出口企业实现出口4.06亿美元，增长35.5%。

（三）项目带动深入实施，城乡建设统筹推进

投资再创历史新高。全社会固定资产投资完成327.18亿元，增长36.6%，增幅创1997年以来新高，其中工业投资完成133.61亿元，增长22%。全市239个在建重点项目完成投资190.5亿元，完成年度计划116.8%。艾默生传动轴承、厨师食品等一批重点项目建成投产，龙厦铁路、厦深铁路、福欣特殊钢等一批重特大项目相继开工建设，厦漳跨海大桥、厦成高速公路海沧至天宝段、福广高速公路复线长泰段、临港燃煤电厂和核电等一批重大项目前期工作加紧推进。

港口开发取得突破。修编《漳州市港口总体规划》，编制《漳州市依港立市暨海洋经济发展规划》。招银港区7号10万吨级码头建成投产，招银四区30万吨大型泊位码头正式动工，招银港区2个15万吨级集装箱码头、后石港区海洋石化25万吨级码头、龙池金帝2个3万吨级专用码头、古雷港区5万吨级石油化工专用码头等加快建设，港尾、古雷、东山等疏港铁路支线前期工作扎实推进。

城乡面貌明显改观。开展滨江南岸、海西城镇群等规划，城乡规划工作进一步加强。投资12.58 亿元用于市区基础设施建设，西洋坪大桥、南昌东路、龙文北路正式通车，投资7.05亿元、全长6.05公里的漳华路基本建成，江滨大道西延伸段基本贯通，中山步行桥启动建设。创卫、创模工作扎实推进，净化绿化美化亮化力度加大，迎宾路十公里路灯等61个“夜景工程”新建改建任务顺利完成，违法占地、违章建设整治效果明显，城市品位进一步提升。县城建设步伐加快，配套功能不断完善，面貌得到明显改观。农村环境整治工程顺利推进，“千村整治、百村示范”、“三清六改”等工程深入实施，第二批“家园清洁行动”镇（村）通过省级抽查验收。

环境保护得到加强。编制《漳州市生态市建设规划》，积极创建绿色社区、绿色学校、绿色企业，加快推进生态文明建设。海洋环境污染整治力度加大，九龙江漳州段水质达标率95.8%，城市环境综合整治定量考核全省第一。节能减排工作取得较好成效，单位生产总值能耗下降2.6%，主要污染物排放量控制在省政府下达指标之内。

（四）民生保障更加有力，社会事业协调发展

人民生活持续改善。城镇居民人均可支配收入14153元，增长13.1%；农民人均纯收入5696 元，增长12.2%，增速为“八五”以来最快。调整提高城市低保、农村低保和优抚标准，全市12.84万城乡低保对象生活得到保障，残疾人等弱势群体基本生活得到妥善安置。新增城镇职工养老保险1.97万人、医疗保险3.5万人。社区卫生服务框架初步形成，新型农村合作医疗全面推开，参合农民305.2万人，参合率84 %。新增就业5.62万人，下岗失业人员实现再就业1.21万人，转移农村劳动力7.92万人，零就业家庭就业问题得到有效解决，城镇登记失业率3.8%。

惠民实事全面落实。31个为民办实事项目投资14.31亿元，完成年度投资计划的150.6%。挂钩帮扶老区村、民族村、贫困村工作深入开展。平和被确认为中央苏区县。农村义务教育“两免一补”政策全面兑现，减免费用8247万元，受惠学生50多万人。建成农村水泥路1067公里，完成年度计划211%，全市99.5%建制村通水泥路。水利建设“六千”工程投资完成年度计划110.4%，防灾减灾体系进一步健全。40所乡镇卫生院得到改造提升，“侨居造福工程”新建住房1554套，纳入规划的124个村级组织活动场所全部建成，农民体育健身工程基本完成。开工建设经济适用房23.5万平方米，市区359户无房户享受廉租房保障。产品质量和食品安全专项整治行动扎实开展，13个100%的目标如期完成。打假及市场整规工作取得新成效。

社会事业健康发展。漳州市获得全国科技进步先进市称号，11个县（市、区）再次全部通过科技进步考核。新增省级高新技术企业25家，高新技术产业产值228.48亿元，增长10.8%。“两基”成果得到巩固，“双高普九”扎实推进，改造农村中小学校舍危房6.41万平方米；整合优化市区小学布局，可扩大招生容量3000多人；高中阶段毛入学率71.2%，提高1.9个百分点；高考本科上线人数首次突破万人大关；新增4所职业技术学院，漳州职业技术学院被列入国家示范性高职立项建设院校。人才强市战略深入实施，新增高级人才1272人。成功举办漳台族谱对接成果展、林语堂国际学术研讨会以及纪念黄道周、沈耀初书画艺术交流活动，土楼申报世界文化遗产顺利通过专家现场评估，闽南文化生态保护实验区正式授牌，南靖被评为全国文物保护先进县。“全民健身与奥运同行”主题活动广泛开展，省农运会获得金牌数居全省第二，谢荔梅等4名运动员入选北京奥运会。低生育水平保持稳定，出生人口政策符合率明显提高，性别比上升势头得到初步遏制，人口自然增长率6.55‰。强化国土资源管理，整治土地2.08万亩，继续保持耕地占补平衡。妇女儿童、老干老龄、民族宗教、外事侨务、统计、物价、史志、档案、地震、人防、气象、国家安全、国防动员、打击走私等各项工作都取得新成绩。

（五）文明法治不断进步，政府建设得到加强

精神文明建设扎实推进。以学习宣传党的十七大精神为重点，加强理论武装工作。以“爱我漳州、从我做起”宣传教育为主线，广泛开展社会主义荣辱观教育，大力实施宣传文化中心户“百千万工程”，组织开展“感动漳州人物”、“文明礼仪进万家”等系列活动，深化文明城市（县城、城区）、文明行业和文明和谐示范村等创建活动，提高110社会服务联动工作水平，不断激发广大市民建设漳州的热情。荣获全国双拥模范城“四连冠”。市公安局强制戒毒所被国务院授予“模范戒毒所”荣誉称号

民主法制建设步伐加快。主动向人大及其常委会报告工作，向政协通报情况，办理人大代表建议156件、政协委员提案298件。加强与工、青、妇等人民团体联系沟通，自觉接受社会监督。“五五”普法活动深入开展，行政复议工作得到加强。深化“平安漳州”建设，开展“平安企业建设年”活动，公众安全感满意率94.3%，综治考核名列全省第一。信访问题解决力度加大，信访总量、集体访批数、越级上访总量均呈下降趋势。加强安全生产管理监督，安全生产四项控制指标全面下降。

政府自身建设不断加强。深化机关效能建设，深入落实“重在提请、主动服务”的工作要求，大力倡导雷厉风行、奋发有为的工作作风，积极推行“三个一线”的工作方法，促进行政提速、服务优化。深入贯彻行政处罚法、行政许可法和公务员法，认真开展行政处罚与行政许可案件评查工作，在全省率先推行规范行政自由裁量权试点工作，依法行政工作取得新成效。认真落实党风廉政建设责任制，加大源头防治腐败工作力度，加强行政监察和审计工作，严肃查处违法违纪行为，政风行风进一步好转。

各位代表，过去的一年，我市在宏观政策趋紧、要素制约增多的情况下，实现了经济社会持续快速发展，多项经济指标增幅超出预期，在全省位次前移。我们深切体会到，政府工作每向前推进一步，漳州事业每取得一项成功，都凝聚着全体代表、各位委员的智慧和力量，饱含着全市人民的心血和汗水。在此，我代表市政府向辛勤工作在各行各业的广大干部群众，向给予政府工作大力支持的人大代表、政协委员、各民主党派、工商联、离退休老同志、各界人士，向为漳州改革发展稳定做出积极贡献的中央、省驻漳单位和驻漳部队、武警官兵，向所有来漳的投资者和建设者，向关心支持漳州发展的港澳台同胞、海外侨胞和国际友人，表示衷心的感谢并致以崇高的敬意！

各位代表，在看到成绩和进步的同时，我们也清醒地认识到发展中还存在不少困难和问题。主要有：经济综合实力不强，重大产业项目需要进一步突破；资源环境约束加剧，经济发展方式需要进一步转变；开放型经济水平不高，漳台合作层次需要进一步提升；新农村建设任务仍然繁重，农民持续增收空间需要进一步拓展；公共产品欠帐较多，社会事业发展需要进一步加强；价格结构性上涨过快，市场调控力度需要进一步加大；维护社会稳定任务依然艰巨，和谐社会建设需要进一步推进；政风行风仍然存在一些不足，政府自身建设需要进一步强化。我们必须高度重视并认真解决这些问题，切实把各项工作做得更好，以不辜负全市人民的期望！

二、2008年工作安排

2008年是全面贯彻落实党的十七大精神的开局之年，是举国上下喜迎北京奥运会的欢庆之年，也是我市加快崛起赶超的关键一年，做好今年工作意义十分重大。我们一定要牢牢把握“四谋发展”实践主题、“四个重在”实践要领、“四个关键”实践要求、“四求先行”实践方向，善于从国际国内大势中把握机遇，善于从宏观政策调整中拓展空间，善于从海西效应扩张中凸显作为，以更宽视野谋划发展，以更活方法破解难题，以更实举措推动工作，着力提升产业支撑力、城乡协调力、发展源动力、民生保障力、文化竞争力和政府公信力，推进漳州在海西建设中奋力先行。

今年政府工作的总体要求是：高举中国特色社会主义伟大旗帜，以邓小平理论和“三个代表”重要思想为指导，认真落实科学发展观，全面贯彻党的十七大以及中央、全省经济工作会议和省十一届人大一次会议精神，积极推进海峡西岸经济区建设，围绕全省建成“两个先行区”目标要求，突出“建设海西、漳州先行”发展主线，深入实施“依港立市、工业强市、开放活市、科教兴市”发展战略，着力强化“项目带动、品牌牵动、统筹互动、形象推动”发展举措，乘势而上促持续，好字当头促提升，以人为本促和谐，提高能力促落实，推动经济社会更好更快发展。

主要预期目标为：生产总值增长13.6%，突破1000亿元；财政总收入增长23%，突破100亿元；规模工业增长24%；农业总产值增长4%；固定资产投资增长25%；社会消费品零售总额增长14%；居民消费价格总水平涨幅控制在4%左右；外贸出口增长10%；实际利用外资增长10%；城镇居民人均可支配收入增长11%，农民人均纯收入增长7%；城镇登记失业率控制在4%以内；人口自然增长率控制在7‰以下；单位生产总值能耗下降2.6%,主要污染物排放量控制在省下达指标之内。在执行中，力争更好更快。

围绕上述要求和目标，必须着力抓好六个方面工作：

（一）坚持量质并举，提升产业支撑力。转变发展方式，调整产业结构，推动产业规模加快壮大、产业结构加速升级。

以基础设施为先导，加快港口开发。统筹规划、合理开发港口岸线、前沿海域和后方陆域资源，提高岸线资源利用效率。加快港口、码头、道路、供水、供电等基础设施建设，夯实项目承接平台，力争临港重大项目引进实现新突破。全力推进招银港区8#、9#泊位及招银航道扩建工程建设，争取厦漳跨海大桥尽快开工，推进港尾铁路支线抓紧立项，做好四区后续仓储区域用地预留与征用。建成古雷开发区供水工程、新杜古延长线，推进杜浔盐场国有土地收储，做好古雷港航道建设项目前期工作，为全面开发古雷港做好准备。抓紧规划连接沿海各港区的滨海一级疏港公路，加快构建山区出海通道。理顺全市港口管理体制，争取国家级东山经济技术开发区扩区和厦门湾北岸政策向南岸延伸，扎实推进石码、冬古、宫口、旧镇四个二类口岸并入一类口岸工作。完善主要港区口岸查验、港务等配套服务设施建设，优化口岸大通关环境，增强综合服务能力。

以优势产业为重点，壮大工业总量。围绕打造海峡西岸先进制造业基地的目标，突出“3+4”优势产业和13个产业集群，继续实施“千百工程”，推进工业总量扩张。抓住福欣特殊钢落户契机，吸引关联项目进驻，扶持帮助凯西、凯景等企业达产扩产，持续扩张钢铁产业规模；扶持金龙、福环、新福达等整车企业扩张，抓紧江淮汽车落户建设，做大矢崎、正兴等一批汽配企业，加快壮大汽车汽配产业集群；推动三菱重工游艇、日产游艇、鸿鹰修造船、华浮造船等项目加快建设，继续引进一批修造船项目，拓展临港修造船产业；加快后石电厂七号机组、大唐风电三期、东山风电二期扩建、大唐新能源研究基地等项目建设，做实国电核电、吴田山抽水蓄能等重大项目前期工作，努力打造全省能源基地。

以自主创新为核心，推进产业升级。加快推进创新型城市建设，实施科技产业建设工程，发展壮大新材料、光电、智能仪表等一批产业基地，开工建设科能仪器科技工业园、富顺科技工业园、钟表工业园等一批产业园区。扶持万利达便携式电脑项目迅速做大，推进灿坤、立达信等企业加快发展，壮大电子信息产业。完善科技创业服务体系建设，建立高新技术创业孵化基地及六个行业技术中心，提升“6·18”项目成果对接能力，深化与国内外知名院校、科研院所合作。加强以高层次、高技能人才为重点的各类人才队伍建设，重视引进海外留学人员、科技领军人才和一线创新人才，壮大一批自主创新型科技示范企业，发展一批知识产权专利示范企业，开发一批专利产品。鼓励有条件的企业牵头或参与国际、国家行业标准的制修订，抢占行业技术制高点。争取全市高新技术企业达150家，高新技术产业产值超300亿元。

以项目带动为龙头，增强发展后劲。妥善应对要素制约，全力抓好重大项目，促进投资总量扩张、项目质量提升。千方百计保障206个市级在建重点项目按计划推进，确保后石电厂七号机组、海安水产等一批大项目建成投产，福欣特殊钢、漳浦台玻、厦深铁路、龙厦铁路等已开工大项目加快建设，厦漳高速扩建工程等新开工大项目按计划开工。更加注重项目前期工作，力促厦成高速（漳州段）、福广高速（长泰段）、枋洋水利枢纽工程等大项目早日开工，全力盯紧石化、汽车等一批能够促进跳跃式发展的重特大项目。

以品牌带动为抓手，提升竞争实力。开展“以质取胜、品牌兴企”专项行动，制定品牌培育奖励政策，建立品牌产品、企业、行业培育目录，加快商标注册代理、质量体系认证等中介组织发展，形成品牌培育工作合力。引导现有中国驰名商标、中国名牌产品争创国际性知名品牌，扶持现有省级品牌创出更多国家级品牌，力争年内新增一批中国名牌产品和中国驰名商标，扩大品牌群体规模。鼓励现有品牌企业实施行业内扩张，注重引进国际性、国家级品牌企业落户漳州，借助外力壮大知名品牌群体。

以扩大消费为目标，加速三产发展。出台优惠政策吸引船代、货代、仓储、配送等企业落户，增加近远洋航线航班，扶持做大原木、粮食、糖等大宗货物物流业务。依托高速公路和铁路，规划建设若干现代物流园区。加快三大商贸广场、六大市场、八大酒店建设，大力发展电子商务、服务外包、创意产业等新型业态。加紧“万村千乡”和“双百”工程建设，繁荣农村市场。认真落实房地产宏观调控政策，促进房地产业持续健康发展。支持金融业稳健发展，为地方经济发展提供有力支撑。科学合理开发旅游资源，扶持东山县争创全国优秀旅游县，支持云洞岩景区、龙佳温泉山庄争创国家4A级景区。深入做好土楼申遗工作，推进土楼文化生态旅游区建设，打响土楼文化旅游品牌。加快滨海火山二期、赵家堡二期、市区明清历史街区等特色旅游景区建设，努力打造“山海旅游圈”精品线路。

以节能减排为手段，促进持续发展。严格节能减排目标责任制，严把项目环评审批，加强生态环境建设，增强可持续发展能力。做好全国第一次污染源普查工作，摸清污染排放情况。加快实施漳州天然气（LNG）一期工程，强力督促二氧化硫重点监控企业安装脱硫设施或使用清洁能源。全面建成8个重点开发区污水处理厂，开工建设10座污水处理厂及配套管网，加快推进垃圾处理产业化。实施重点排污单位在线监控，严肃查处违法排污企业。推进重点流域、重点区域、重点海域环境整治，实施九龙江流域生态保护工程，着力抓好饮用水源地流域环境治理保护，确保饮用水安全。认真贯彻节能法，推进节能降耗工程，综合运用产业政策、环境容量、差别电价等措施，推广节能环保新技术、新工艺、新设备，淘汰落后生产能力。推行清洁生产，鼓励生产使用节能、节水、节材产品。加快推进循环经济示范工程，完善循环经济促进机制。严格国土资源管理，继续保持耕地占补平衡，积极引导建设节地型工业园区，严格落实建设用地投资强度和准入规定，有效促进节约集约用地。

（二）坚持以城带乡，提升城乡协调力。按照城乡一体化发展的新要求，统筹城乡发展，强化互动共荣，加快城市现代化、农村城镇化进程。

促进中心城市扩张辐射。按照“拉大框架、跨江发展，路网先行、重点突破，打造亮点、提升品位”的思路，进一步加大城市建设力度，持续完善城市功能，努力建设宜居城市。高站位高起点规划城市。做好中心城区拓展规划，将市区100平方公里规划扩大到300平方公里，编制城市建筑色彩及设计风格规划和漳州南站周边地块城市设计及控制性详规，精心设计城市亮点片区和精品建筑，引领城市科学合理建设。坚持组团式、功能化发展，科学规划总部经济区、物流集中区、商贸中心区、休闲娱乐区等功能布局。高品位高标准建设城市。建设好每一条路、每一幢楼、每一个工程，体现特色，形成亮点，提升品位。扎实推进中山桥、南江滨路芗城段、延安南片区、南山景区、滨江公园建设，加快形成一江两岸、沿江发展的城市格局。开工建设零号路、腾飞路西段、大学西路中段、新浦东路、六号路、十三号路、毅达新城西侧南侧道路，续建西环城路、江滨路西延伸段、江滨路防洪堤改造、瑞京路西段、钟法路南段、西洋坪北路，完善城市路网，壮大城市体量。高效能高水平管理城市。深化国家级卫生城市创建工作，强化街道、社区基础作用，推进城市精细化、人性化管理。制定实施物业管理办法，提高物业管理水平。改进道路交通管理，缓解市区交通拥堵现象。加大对城郊地带的整治力度，切实改变城郊结合部的环境面貌。加快市政公用事业社会化改革步伐，提高城市资源的配置效益。

促进县域经济竞相发展。进一步发挥县域主体作用，激发县域经济发展活力，促进县域经济加速跃升。做大做美县城。完善县城总体规划和专项规划，构建商贸区、住宅区、科教区、休闲区组团发展新格局。推进县城基础设施建设，扩大路网框架，壮大县城体量。推进县城净化绿化美化亮化，加强社会事业配套建设，提升县城人气商气。推进九龙江沿岸、沿海地带和高速公路沿线城镇密集区建设，做好厦深、龙厦铁路沿线城镇建设规划，促进现代城镇群加快形成。做优做强园区。做好园区发展规划和产业集群规划的衔接，用好国家有关部委支持海峡西岸开发区扩区升级及改变区位等政策，整合壮大提升产业园区。建立健全以市场运作为主的开发机制、以完善服务体系为重点的协调推进机制，积极吸纳各类资本投资建设产业园区。推进园区土地集约经营、产业链式延伸、项目集群组合、资源循环利用，努力形成一批配套程度高、集聚效应强的特色产业，不断提高产业园区投入强度和产出水平。规划建设外来工公寓，改善外来工生活条件。做活做实节会。改造扩建花博园，扩大展厅规模，完善配套设施，努力办好第十届花博会，多举办各类农产品展销会，把花博园打造成海峡西岸农产品展示平台和采购中心。继续支持各县依托产业基础和文化特色举办各种节会，以节会促进氛围营造、项目引进、发展提速。

促进新农村建设走在前列。坚持工业化致富农民、城镇化带动农村、产业化提升农业，深入实施新农村建设规划，推动新农村建设走在全省前列。把发展现代农业作为首要任务。突出“产业化、规模化、特色化、外向化、品牌化”的发展方向，用现代理念引领农业发展，增强农业综合生产能力。稳定粮食生产，确保粮食安全。推进水果、蔬菜、水产、食用菌、花卉、畜牧、茶叶、林业等八个产业板块建设，做好花卉产业发展规划，加快闽荷花卉合作项目建设，提升与荷兰花卉产业合作层次。鼓励发展“一县一业、一县一品”，支持一批农产品深加工基地建设。加大对农业产业化龙头企业扶持力度，争取培育两个以上年销售额超10亿元的龙头企业。加快科技兴农步伐，健全农业技术推广体系，积极推广农业“五新”，用现代技术改造农业。筹建闽台农业合作食用菌产业园区，发展农产品对台物流市场，争取外向型农业新突破。把增加农民收入作为核心目标。广辟增收渠道，不断拓展农民持续增收空间。加强农产品质量安全检测监管体系建设，大力发展无公害农产品、绿色食品、有机食品，培育一批品牌农产品，增加农民经营性收入。实施新型农民科技培训和劳动力转移培训“阳光工程”，扩大劳务输出，增加农民工资性收入。扶持建设一批具有产业支撑、辐射力较强的批发市场，发展农民专业合作社、农村服务组织和农产品行业协会，提高农民进入市场的组织化程度。继续加大扶贫开发和“造福工程”实施力度，做好库区移民后期扶持工作，支持老区、边区、少数民族地区加快发展，改善贫困村生产生活条件。把完善基础体系作为关键依托。按照中央“三个明显高于”的要求，进一步加大对农业农村的投入，确保各项强农惠农政策落实到位。继续实施水利建设“六千”、广播电视电信“村村通”等工程，续建延伸农村路网工程，改造重建普通公路危桥，推进生态林、沿海防护林建设，加快一批渔港建设步伐。完善农业防灾减灾体系，加快实施海洋防灾减灾“百千万工程”，抓好动植物疫情防控、防汛抗旱、渔业生产安全、森林防火等工作。鼓励设立新型农村银行机构，创新“三农”信贷担保体系，推进农业政策性保险，改善农村金融服务。加强村级“六大员”和基层农技推广队伍建设，增强服务“三农”能力。深化村务公开和民主管理工作，完善党员干部驻村任职工作机制，促进农村和谐发展。把农村环境整治作为重要内容。加快建制镇和中心村建设规划编制工作，抓好新农村试点示范村建设，推动有条件的村庄发展新农村社区。深入开展“创绿色家园、建富裕新村”和“环境优美乡镇”、“生态村”创建活动，实施以“家园清洁行动”为载体、以“三清六改”为主要内容的村容村貌整治工程，突出抓好农村垃圾清运、禽畜养殖业污染治理、沼气推广和镇村“四旁”绿化，着力改善农村环境卫生。

（三）坚持改革开放，提升发展源动力。坚定不移地深化改革、扩大开放，为加快漳州发展释放新能量、增添新动力。

漳台合作要先行一步。积极主动开展工作，努力使漳州成为中央惠台政策的先行地区。认真研究台湾新一轮产业转移动态，瞄准石化、电子、信息、汽车、船舶等产业，创造条件承接产业外移。跟踪了解台湾百强制造业和服务业企业的投资合作意向，找准时机对接招商，努力实现台湾“双百”企业引进新突破。发挥台商协会桥梁作用，加强与台湾工商社团、行业中介组织联系与交流，探索建立长效协作机制。深化漳台农业合作，大力引进台湾农业先进技术、优良品种和农产品深加工企业。做大台湾农民创业园，吸引更多台湾农民来漳创业。抓住中央不断实施和充实惠及广大台湾同胞政策措施的有利时机，探索建立台湾农产品进入我市的便捷机制。推动赴台煺钩Ｌ???欢侠┐蠖蕴?骋住?/SPAN>

利用外资要有新突破。深化漳台港澳侨合作，加强对欧美、日韩、东南亚等国家的招商，形成全方位招商新格局。以三大招商活动为主要载体，创新招商机制，提升招商水平，全面拓展利用外资渠道。以规划引领招商，按照生产力布局和产业规划优选招商重点和方向，引导产业对接。以资源引领招商，发挥港口、土地等资源优势，瞄准重大投资主体，承接项目落户。以环境引领招商，加快完善基础设施，打造一流投资环境，吸引企业入驻。突出专业招商，委托专业机构进行推介运作，提升招商的针对性与实效性。突出产业链招商，围绕优势产业和现代服务业，促进上下游产业延伸和集聚。突出以商引商，鼓励引导现有企业在招商引资中发挥更大作用，形成招商引资的群体效应。把“引进来”和“走出去”更好结合起来，不断拓展对外开放的广度和深度。

外贸出口要持续增长。鼓励企业开展各类质量体系、环境体系和产品认证，推行标准化生产，不断提高产品质量，新增一批国家免检产品。帮助企业创立自主出口品牌，积极申报科技兴贸创新基地，争取设立国家光电技术产业出口创新基地。完善挂钩服务制度，帮助重点企业解决困难和问题，落实扶持中小企业出口政策，支持各类企业做大出口规模。鼓励企业开展进口业务，扩大国外先进技术、关键设备和重要原材料进口。发挥海峡两岸（福建东山）水产品加工集散基地优势，争取设立东山东海岸保税物流中心，吸引国内外水产品到我市仓储、加工和分销。

民营经济要加快发展。落实鼓励和支持民营经济发展的各项政策，深入实施“回归工程”，广泛激发民间创业热情。健全民营企业服务体系，深化创业辅导、融资担保、技术支持、人才培训、用工保障、信息咨询、市场开拓等服务，扶持民营企业上规模、上水平、上档次。加强区域经济协作，积极承接珠三角、长三角及周边发达地区产业转移，争取一批知名度高、影响力大的民营企业落户漳州。

体制改革要配套推进。深化国有企业改革，积极推进力佳、工业安装公司等企业改制重组。加强国有资产监管，健全经营业绩考核体系，完善公司法人治理结构。高度重视企业上市工作，制定出台激励政策，建立上市企业资源库，扶持培育一批企业上市直接融资。深化投融资体制改革，稳步推进事业单位改革，深入推进公共资源市场化配置改革。推进行政管理体制改革，加强社会管理和公共服务职能。健全公共财政体系，深化部门预算、国库集中支付改革，规范政府采购行为。深化农村综合改革，推进土地承包经营权有序流转，妥善化解农村义务教育债务，深化集体林权确权发证工作，推进海域使用权市场化配置，加快水利工程管理体制改革。

（四）坚持为民惠民，提升民生保障力。围绕“五有”目标，集中更多新增财力用于改善民生，实施一批为民办实事项目，让广大群众得到更多实惠。

集中财力、增加投入，促进教育事业发展。坚持优先保障教育投入、优先解决教育问题、优先配强教师队伍。实施《漳州市中小学布局调整意见》，加大教育资源整合力度，努力改善办学条件，促进义务教育均衡发展。继续推进农村中小学危房改造和现代远程教育工程，建设一批设施完善的农村寄宿制小学和初中校。免除城市义务教育阶段学生学杂费，免费为农村义务教育阶段学生提供教科书，补助农村义务教育阶段寄宿生生活费。完善困难学生资助体系，保障经济困难家庭平等接受教育，关注农村留守儿童成长，严格控制初中生辍学。加快普及高中阶段教育，重视发展学前教育，扶持办好特殊教育，大力发展职业教育，支持发展高等教育。鼓励和规范社会力量兴办教育。

创业带动、帮扶促动，千方百计扩大就业。认真贯彻劳动合同法和就业促进法，完善鼓励和支持自主创业、自谋职业优惠政策，抓好就业再就业政策落实，新增城镇就业岗位5.2万个，帮助1.2万名下岗失业人员实现再就业，重点解决“零就业”家庭、高校毕业生、“4050”下岗失业人员、被征地农民就业再就业问题。加快建立县、乡、村三级农村劳动力转移就业和培训服务网络，实行农村劳动力“四免一补”就业服务，把进城务工农民纳入公共就业服务体系，力争转移农村富余劳动力7.7万人，培训农民工4万人。

政府主导、社会参与，完善社会保障体系。继续巩固“两个确保”，确保基本养老金和失业金按时足额发放。推进新型农村养老保险制度改革，探索建立被征地农民养老保险制度，维护失地农民的社会保障权益。完善新型农村合作医疗运行管理机制，提高补偿标准，简化报销手续。扩大城镇职工基本医疗保险覆盖面，妥善解决困难企业职工医疗保险历史遗留问题。推进农民工参加工伤保险，维护伤病残农民工合法权益。积极开展机关事业和企业生育保险工作，促进生育保险工作全面发展。继续扩大城乡低保覆盖面，进一步提高低保补助标准。抓紧成立市慈善总会，积极开展“红十字”人道救助活动。鼓励引导社会力量兴办社会福利事业，推进社会福利社会化。加强老龄工作，建设社会福利中心和一批农村敬老院，完成漳州市老年爱心护理院建设。积极推进各项优抚安置政策的完善和落实，保障优抚对象合法权益。扩大农房保险，推行渔工责任保险，开展农村自然灾害公众责任保险，提高灾害应急救助能力。加快社会保障性住房建设，完善廉租房制度，确保完成23.5万平方米经济适用房建设任务，认真解决中低收入家庭住房困难问题。完成“侨居造福工程”。

预防为主、体系保障，提高群众健康水平。做好重大疾病特别是传染病的防控，提高疫情应急处置能力和突发公共卫生事件快速反应能力。加大政府投入，完善覆盖城乡的公共卫生服务体系、医疗服务体系、医疗保障体系、药品供应保障体系。扶持县级医院发展，改造提升15所乡镇卫生院和15所社区卫生服务中心。加强村卫生所建设，消灭49个卫生所空白村。加强产品质量和食品安全监管机构建设，形成长效监管机制，确保群众吃上安全的食品、用上放心的药品。健全人口计生工作新机制，继续稳定低生育水平，提高出生人口素质，全市人口出生率控制在12.6‰以下，出生人口政策符合率达93%以上。

完善管理、综合治理，维护社会和谐稳定。重视发展和培育社会组织，健全基层社会管理机制，提高社会组织度和融合度。加强公民意识教育，深入推进“五五”普法，在全社会形成自觉学法、用法、守法的良好氛围。认真做好人民群众来信来访工作，积极主动开展矛盾纠纷排查调处工作，有效预防和化解各类社会矛盾。深入推进“平安漳州”建设，加强社会治安综合治理，严厉打击各类刑事犯罪，确保群众对社会治安满意率达90%以上。健全突发事件应急机制，提高有效应对突发事件的能力。严格落实安全生产责任制，扎实开展“隐患治理年”活动，严防各类重特大事故发生。同时，认真做好第二次全国经济普查工作，加强妇女儿童、老干老龄、民族宗教、外事侨务、统计、物价、档案、气象、防震等工作，做好国家安全、国防动员、支前双拥、打击走私等工作。

（五）坚持突出特色，提升文化竞争力。发挥地方文化优势，创作更多文化精品，更好满足人民群众精神文化需求。

深入开展精神文明创建活动。深入开展“八荣八耻”教育，大力弘扬“三平”精神，持续深化“诚信漳州”建设。进一步加强青少年思想道德教育，促进未成年人健康成长。广泛深入开展群众性精神文明创建活动，提升文明县城、文明村镇、文明社区、文明单位、文明窗口的创建水平。继续深化“文明礼仪进万家”、“婚育新风进万家”活动，不断掀起“迎奥运、讲文明、树新风”活动热潮。扎实推进农村移风易俗、“六进农家”等活动，促进农村精神文明建设上新水平。

建立健全公共文化服务体系。加大财政对公益性文化事业的投入力度，扶持县（市、区）图书馆、文化馆、博物馆“三馆”建设，推进乡镇文化站和文化中心户建设，开展文艺、图书、电影等农村流动服务活动，办好省第二届农民电影节。弘扬科学精神，普及科学知识。突出“全民健身与奥运同行”主题，加强全民健身服务网络建设。启动筹备2009年第十一届市运会，积极备战第十四届省运会。

大力保护发掘特色文化资源。推进漳州闽南文化生态保护实验区建设，开展第三次全国文物普查工作，做好第二次非物质文化遗产普查发掘，加快第二轮续志工作，扶持保护传统工艺、民间艺术、民俗文化、老字号和名人故居等重要文化资源。深入发掘对台文化资源，规划建设开台文化公园，打响“祖地”品牌，推动漳台文化交流和民间交往深入开展。大力拓展传统木偶艺术、芗剧（歌仔戏）、潮剧舞台表演艺术的创新路子，促进特色文化产业发展繁荣。积极培育文化市场主体，扶持文化骨干企业发展。加快新闻出版和广电事业的改革与发展，加大文化市场整顿力度，加强网络文化建设与管理。

（六）坚持实干实效，提升政府公信力。进一步加强政府自身建设，推进政府管理创新，立足实干，务求实效，建设对人民负责的政府，不断提升政府的公信力。

解放思想，创新理政。勇于站在解放思想的最前列，坚决破除一切落后于时代步伐、滞后于形势变化、有碍于经济发展的思想观念，牢固树立顺应时代潮流、符合客观规律、能够推动发展的思想观念，向解放思想要动力、要活力、要生产力。对有利于促进发展、造福百姓的事，不观望、不等待、不推诿，大胆闯、大胆做、大胆试。对有利于推进改革、破解难题的事，少评论、少设障、少指责，多支持、多理解、多宽容。

注重运作，高效施政。谋深谋细促运作，从更高站位、更高层次、更高水平上谋划思路、谋划项目、谋划对策，突破制约发展的各种瓶颈，化解影响发展的各种矛盾。立说立行促落实，对上级做出的决策部署要在最短时间内贯彻到位、落实到位，对工作中出现的困难问题要在最短时间内化解到位、解决到位。合心合力促协调，向上要主动沟通汇报、争取更多支持，横向要理顺权责关系、加强配合协作，向下要及时指导帮助、强化督促检查。问效问责促执行，深化效能建设，强化绩效考核，严格行政问责，确保政令畅通无阻、政务高效运作。

规范行为，依法行政。全面推进依法行政，健全政府工作规则，完善行政决策程序，深入开展规范行政自由裁量权工作，不断深化行政审批制度改革，推进行政服务中心和公共资源交易中心高效运作。认真执行人大及其常委会的决议，自觉接受人大的法律监督、工作监督和政协的民主监督，积极办理人大代表的建议、意见和政协委员的提案。加强与各民主党派、工商联、无党派人士的联系，广泛听取他们的意见和建议。认真实施政府信息公开条例，加强政府网站建设，大力发展电子政务，提高政府工作透明度。

敢抓敢管，从严治政。各级政府各部门领导要勇于担当，敢抓敢管，管不落实之人，抓不落实之事，让每个人都负起责任，让每项工作都有效落实。切实加强政府系统反腐倡廉建设，健全惩治和预防腐败体系，更加注重治本，更加注重预防，更加注重制度建设，严防权力滥用，严查商业贿赂，严惩腐败分子，确保政务规范、政事清明、政风端正。坚持厉行节约，反对铺张浪费，建设节约型政府。深入贯彻公务员法，加强对公务员的教育、培训和监督，着力建设一支政治强、业务精、作风硬的公务员队伍，树立为民、务实、清廉的良好形象。

各位代表，漳州发展正处在一个关键节点，进入一个上升时期。我们要更加紧密地团结在以胡锦涛同志为总书记的党中央周围，在市委的正确领导下，紧紧依靠全市人民，振奋精神、高昂激情，拓展作为、奋力先行，为圆满完成全年目标任务、加快实现漳州崛起赶超而努力奋斗！
